# Perceived Stress Levels, Chemotherapy, Radiation Treatment and Tumor Characteristics Are Associated with a Persistent Increased Frequency of Somatic Chromosomal Instability in Women Diagnosed with Breast Cancer: A One Year Longitudinal Study

**DOI:** 10.1371/journal.pone.0133380

**Published:** 2015-07-15

**Authors:** Noran Aboalela, Debra Lyon, R. K. Elswick, Debra Lynch Kelly, Jenni Brumelle, Harry D. Bear, Colleen Jackson-Cook

**Affiliations:** 1 Department of Human & Molecular Genetics, Virginia Commonwealth University, Richmond, Virginia, United States of America; 2 Family and Community Health Nursing, School of Nursing, Virginia Commonwealth University, Richmond, Virginia, United States of America; 3 Department of Pathology, Virginia Commonwealth University, Richmond, Virginia, United States of America; 4 Division of Surgical Oncology, Virginia Commonwealth University, Richmond, Virginia, United States of America; 5 Massey Cancer Center, Virginia Commonwealth University, Richmond, Virginia, United States of America; Taipei Medical University, TAIWAN

## Abstract

While advances in therapeutic approaches have resulted in improved survival rates for women diagnosed with breast cancer, subsets of these survivors develop persistent psychoneurological symptoms (fatigue, depression/anxiety, cognitive dysfunction) that compromise their quality of life. The biological basis for these persistent symptoms is unclear, but could reflect the acquisition of soma-wide chromosomal instability following the multiple biological/psychological exposures associated with the diagnosis/treatment of breast cancer. An essential first step toward testing this hypothesis is to determine if these cancer-related exposures are indeed associated with somatic chromosomal instability frequencies. Towards this end, we longitudinally studied 71 women (ages 23-71) with early-stage breast cancer and quantified their somatic chromosomal instability levels using a cytokinesis-blocked micronuclear/cytome assay at 4 timepoints: before chemotherapy (baseline); four weeks after chemotherapy initiation; six months after chemotherapy (at which time some women received radiotherapy); and one year following chemotherapy initiation. Overall, a significant change in instability frequencies was observed over time, with this change differing based on whether the women received radiotherapy (p=0.0052). Also, significantly higher instability values were observed one year after treatment initiation compared to baseline for the women who received: sequential taxotere/doxorubicin/cyclophosphamide (*p*<0.001) or taxotere/cyclophosphamide (*p*=0.014). Significant predictive associations for acquired micronuclear/cytome abnormality frequencies were also observed for race (*p*=0.0052), tumor type [luminal B tumors] (*p*=0.0053), and perceived stress levels (*p*=0.0129). The impact of perceived stress on micronuclear/cytome frequencies was detected across all visits, with the highest levels of stress being reported at baseline (*p *=0.0024). These findings suggest that the cancer-related exposome has an impact on both healthy somatic cells and tumor cells, and may lead to persistent chromosomal instability. In addition, stress was a significant predictor of chromosomal instability; thus, interventions that aim to reduce stress may reduce acquired soma-wide chromosomal instability for cancer survivors.

## Introduction

Innovations in treatment approaches for women with early-stage breast cancer, such as the use of taxanes and anthracylines in sequential, standard or dose dense regimens (along with radiotherapy and/or hormonal antagonists), have resulted in improvements in survival rates [1,2]. Unfortunately, these improvements in survival rates have been accompanied by reports from many survivors of the acquisition and persistence of adverse side effects, which include (but are not limited to): anxiety [3], depression [3], fatigue [4], cognitive dysfunction [5], sleep disturbance [6], and pain [7,8], which we categorize as “psychoneurological symptoms” (PNS). For some survivors these side effects are short-term and limited to the treatment phase. However, long-term side effects can persist for years after treatment and result in reduced quality of life for cancer survivors [9–11]. The biological basis underlying the genesis of these long-term adverse side effects is not known, with no clear consensus regarding a biological mediator(s) yet emerging [12–15]. Furthermore, it is not known if these acquired PNS are associated with the treatments, the perceived stress that accompanies a diagnosis of cancer and its treatment, the cancer itself, or a combination of exposures to biological and psychological factors.

Given that the acquired biological alteration(s) leading to the development and persistence of these adverse side effects must provide a means to be “remembered” or retained for months/years, we have hypothesized that these PNS arise, at least in part, from acquired somatic genetic or epigenetic alterations [16]. One such potential genetic alteration is somatic chromosomal instability. Chromosomal instability is defined as “a constitutional gain or loss of whole chromosomes or fractions of chromosomes” [17]. Soma-wide chromosomal instability could result in the development of a clonal population(s) of cells having a genetic imbalance. Furthermore, increased non-clonal chromosomal instability could alter the function of somatic cells through aberrant gene expression. Evidence supporting a contributing role for acquired somatic chromosomal instability in neurological and psychological conditions has been noted in other health conditions similar to those experienced by breast cancer survivors and includes (but is not limited to) studies of people with chronic pain [18–23], age-related cognitive decline [24], mild cognitive dysfunction [25], fatigue [18], and stress exposure [26]. Furthermore, telomere shortening, which has consistently been shown to lead to increased frequencies of chromosomal instability, has been associated with the development of several PNS (reviewed in [27,28]); however, additional studies are warranted to further evaluate these potential biological relationships.

**Fig 1:**
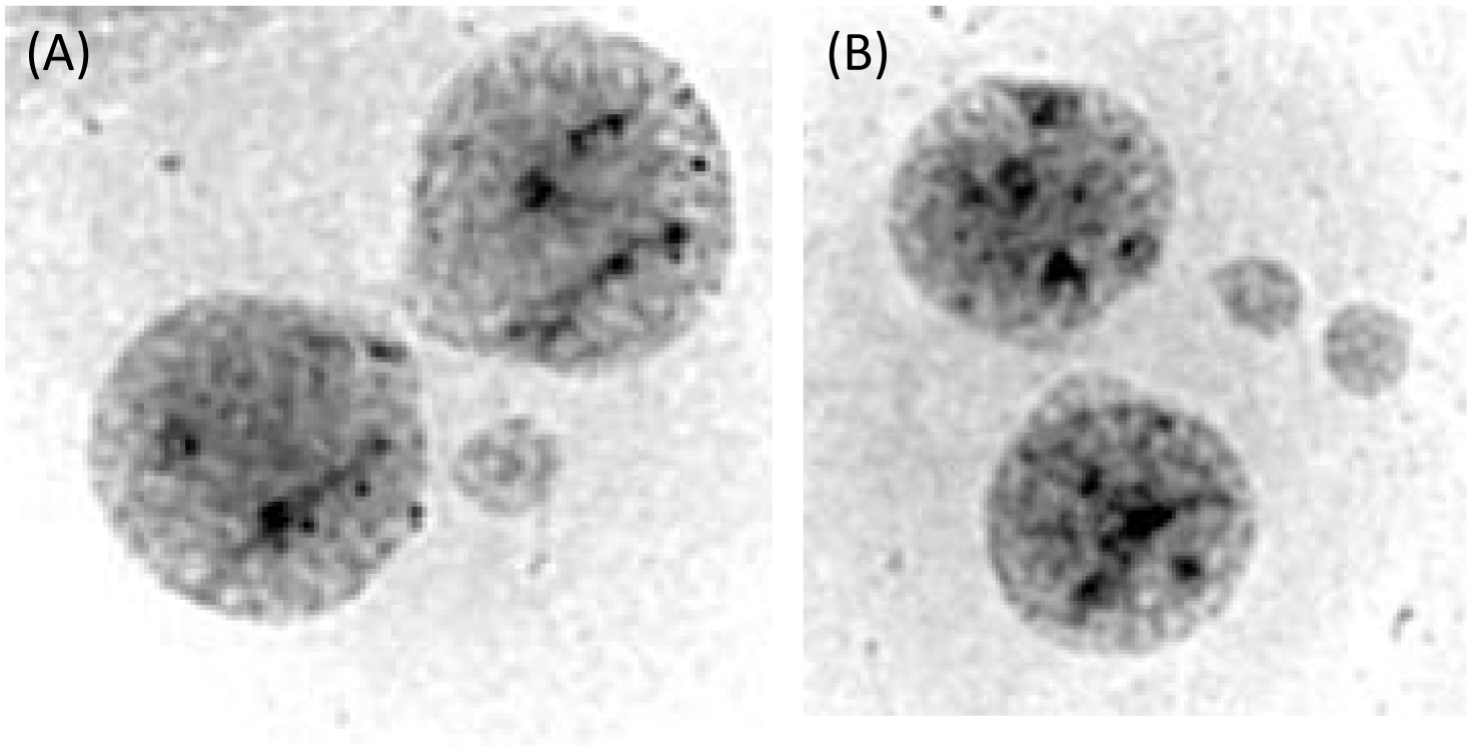
Examples of giemsa stained binucleates with MN. Mitotic cells were blocked at cytokinesis (but did complete karyokinesis) to result in binucleates. In the figure to the left (A) a single micronucleus is present. In the right figure (B) 2 micronuclei have been excluded from the parental cell.

An efficient means for quantifying acquired chromosomal instability associated with exposures is the cytokinesis-block micronucleus (CBMN) assay [29]. Briefly, a micronucleus/micronuclei (MN) is/are a small chromatin-containing structure(s) that is juxtaposed to the main nucleus or daughter binucleates following the completion of mitosis (Fig 1). MN can arise as a result of numerical chromosomal abnormalities (e.g. whole chromosomal lagging or malsegregation at mitosis) or from structural chromosomal abnormalities (e.g. the failure of an acentric fragment or dicentric chromosome to segregate at mitosis)[30]. This relatively high-throughput methodology allows for the assessment of a large numbers of cells, thereby enhancing one’s ability to accurately quantify even low levels of chromosomal instability. In addition, this technique is not readily influenced by technical artifact (e.g., it allows for minimization of *in vitro* growth selection since only one round of cell division is completed *in vitro*). Moreover, this method overcomes several of the challenges experienced when scoring exposure-related chromosomal instability using conventional metaphase chromosomal studies, such as artifactual chromosome “loss” due to cell breakage at harvesting or slide making.

One of the most effective means for assessing the short-term and long-term impact of cancer therapy on normal cells is to longitudinally evaluate patients and compare baseline biological values to those measured during treatment and into recovery. Thus, we initiated a longitudinal study to test the hypothesis that attributes of the tumor cells, exposure to chemotherapy, radiation, and/or perceived stress, lead to an increased frequency of somatic cell chromosomal instability that persists beyond the time of treatment for women diagnosed with breast cancer. Determining if these exposures lead to acquired chromosomal instability is a necessary first step for assessing its potential role in the acquisition/persistence of PNS associated with breast cancer and its treatment.

## Materials and Methods

### Ethics Statement

Human subjects research was approved by the Virginia Commonwealth University IRB (protocol number HM 13194). Written documentation of informed consent was obtained from all research participants.

### Study Participant Ascertainment and Specimen Collection

A total of 77 women with early stage (I to IIIA) breast cancer, who ranged from 23 to 71 years of age, were ascertained through 5 regional cancer centers in Central Virginia. To identify potential study participants, each site had a study coordinator screen patients for eligibility. The eligibility criteria were: (1) an age of 21 years or older; (2) a diagnosis of early stage breast cancer with a scheduled visit to receive chemotherapy; and (3) female gender (males were excluded since too few male participants were available for study). Exclusion criteria were a history of: (1) a previous cancer, or chemotherapy; (2) a diagnosis of dementia; (3) active psychosis; or (4) immune-related diagnoses (e. g. multiple sclerosis; systemic lupus erythematosus). After providing informed consent (VCU IRB #HM 13194), participants were enrolled and the first study visit was scheduled prior to the initiation of chemotherapy. The four time points for evaluation in this longitudinal study were: (1) Visit 1 (baseline), which occurred prior to chemotherapy (but following surgery for the majority of participants); (2) Visit 2, which was scheduled prior to the fourth cycle of chemotherapy; (3) Visit 3, which was scheduled approximately 6 months following the initiation of chemotherapy, at which time a subset of women received radiotherapy; and (4) Visit 4; which was scheduled approximately 1 year following the initiation of chemotherapy. A peripheral blood sample was collected at each visit by venipuncture or through an existing access device and transported to the cytogenetics laboratory. The specimens were coded prior to their delivery to the lab to ensure that the cytogeneticists were unaware of the clinical history or therapy status of each participant at the time of sample processing and evaluation.

### Chromosome Instability Methodology

#### Scoring of Micronuclei, Buds, and Bridges

Chromosomal instability levels were quantified for each specimen using the cytokinesis-block micronucleus (CBMN) and cytome assay [29]. Leukocytes, which were isolated using Histopaque-1077 (Sigma), were established in culture according to standard techniques [31]. Briefly, following their mitogenic stimulation using phytohemaglutinin (PHA), lymphocytes were arrested at cytokinesis by adding cytochalasin B to the cells 44 hours after the cultures were initiated. The cells were harvested 72 hours after culture initiation and slides prepared (2 per specimen) as described previously [32].

Micronuclei, buds, and/or bridges were visualized following giemsa staining (4% Harleco Giemsa solution) and identified according to the criteria established by Fenech [29, 33]. The proportion of abnormalities was calculated by adding the values obtained from two replicate scores (1000 binucleates/mononucleates were evaluated from each of two slides for a total of 2000 binucleates/mononucleates per study participant). The total number of binucleates and mononucleates with abnormalities was then divided by the total number of cells scored to obtain the frequency of chromosomal instability.

#### Scoring of Nuclear Division Cytotoxicity Index

Given that breast cancer treatments might also contribute to differences in nuclear proliferation/viability, the nuclear division cytotoxicity index (NDCI) was determined using the scoring and calculation criteria of Eastmond and Tucker [34], as adapted by Fenech [31]. Briefly, the NDCI was calculated using the following formula:

NDCI = [Ap+Nec+M1+2(M2)+3(M3)+4(M4)]/N

where Ap = the number of apoptotic cells; Nec = the number of necrotic cells; M1; M2; M3; and M4 = the number of cells having 1, 2, 3, or 4 nuclei, respectively; and N = total number of cells scored (viable as well as non-viable).

### Perceived Stress Assessment

The degree to which individuals perceived their lives to be stressful was measured using the Perceived Stress Scale (PSS; V.9/21/09) [35]. This 10-question self-report measure indicates how often individuals found their lives to be unpredictable, uncontrollable, and overloaded in the past month. Each question has five possible Likert scale responses (never, almost never, sometimes, fairly often, and very often). Of the 10 total responses on the PSS, 6 were “negative” responses and were given the following numerical value: never = 0; almost never = 1; sometimes = 2; fairly often = 3; and very often = 4. The remaining 4 positive items in the PSS were scored in a reversed format according to standard approaches (i.e. never = 4…very often = 0) [36, 37]. All 10 response values were then summed to calculate the participants’ total perceived stress score, with higher scores indicating higher levels of perceived stress [35].

### Recognition of Treatments and Breast Tumor Characteristics

Medical records were used to determine the type and duration of the chemotherapy regimens, as well as breast tumor attributes of patients. Medical records also revealed if the participants received radiotherapy in addition to chemotherapy. Based on pathology reports, the tumors were categorized as either: (1) luminal A (human epidermal growth factor receptor 2 [HER2] -; estrogen receptor and/or progesterone receptor +; no information was available about Ki67 values); (2) luminal B (HER2+; estrogen receptor and/or progesterone receptor +; no information was available about Ki67 values); (3) HER2 positive (HER2+; estrogen receptor -; progesterone receptor -); or (4) triple negative (HER2-; estrogen receptor -; progesterone receptor -) [38–42].

### Demographic, Lifestyle, and Other Health Information

Demographic information for the study participants was collected by self-report using a questionnaire format. Demographic variables evaluated included age, race, and income. Similarly, lifestyle factors [nutritional practices (eating vegetables/fruit); smoking status, and alcohol consumption], and menopausal status (pre- or post-) were evaluated by self-report. However, medical records were used to determine body mass index.

### Statistical analysis

Descriptive statistics of MN/cytome frequencies were computed for the demographic, tumor characteristic, treatments, and stress variables noted above. All data were examined graphically to determine their distributional properties. It was anticipated that the MN/cytome frequencies would follow a log-normal distribution, as is typically seen with MN frequency data [43]. However, because of the relatively high number of MN in this population, the MN frequencies were approximately normally distributed. Demographic, tumor characteristics and treatment variables in Tables 1–3 were compared using chi-square tests for categorical data and a two-sample t-test for continuous data when looking for differences in Race (African American vs. Caucasian).

**Table 1:** Demographic, health and lifestyle findings in study participants receiving chemotherapy for breast cancer.

Demographic Variables^1^	African American N = 22				Caucasian N = 49				
	TAC	TC	TCH	AA Total	TAC	TC	TCH	C. Total	Study Total
	10 (45%)	6 (27%)	6 (27%)	22 (31%)	29 (59%)	15 (31%)	5 (10%)	49 (69%)	71 (100%)
**Income** ^**1**^									
Less than $30,000	5 (23%)	4 (18%)	3 (14%)	12 (55%)	6 (12%)	1 (2%)	0	7 (14%)	19 (27%)
$30,000-$59,999	3 (14%)	2 (9%)	3 (14%)	8 (36%)	6 (12%)	1 (2%)	0	7 (14%)	15 (21%)
$60,000-$89,999	1 (5%)	0	0	1 (5%)	6 (12%)	7 (14%)	3 (6%)	16 (33%)	17 (24%)
$90,000+	1 (5%)	0	0	1 (5%)	11 (22%)	6 (12%)	2 (4%)	19 (39%)	20 (28%)
**Age** ^**2**^									
	44.0 (3.4)	47.0 (2.5)	50.5 (2.9)	46.6 (1.9)	52.2 (1.7)	57.1 (2.6)	50.2 (6.4)	53.5 (1.5)	51.3 (1.2)
**Menopausal Status** ^**1**^									
Pre- or Peri-	7 (32%)	3 (14%)	2 (9%)	12 (55%)	11 (22%)	5 (10%)	3 (6%)	19 (39%)	31 (44%)
Post-	3 (14%)	3 (14%)	4 (18%)	10 (45%)	18 (37%)	10 (20%)	2 (4%)	30 (61%)	40 (56%)
**BMI** ^**2**^									
	32.0 (2.7)	29.8 (2.5)	35.6 (6.3)	32.4 (2.1)	30.1 (1.5)	26.8 (0.9)	37.3 (4.7)	29.8 (1.1)	30.6 (1.0)
**Nutrition** ^**2**^ ^,^ ^**3**^									
	2.6 (0.18)	2.2 (0.21)	2.4 (0.25)	2.4 (0.12)	2.7 (0.11)	2.9 (0.13)	2.7 (0.30)	2.8 (0.08)	2.7 (0.07)
**Smoking Status** ^**1**^									
Yes	2 (9%)	3 (14%)	2 (9%)	7 (32%)	4 (8%)	2 (4%)	1 (2%)	7 (12%)	14 (20%)
No	8 (36%)	3 (14%)	4 (18%)	15 (68%)	25 (51%)	13 (27%)	4 (8%)	42 (88%)	57 (80%)
**Ethanol Use** ^**1**^									
Yes	3 (14%)	1 (5%)	2 (9%)	6 (27%)	19 (39%)	11 (22%)	4 (8%)	34 (69%)	40 (56%)
No	7 (32%)	5 (23%)	4 (18%)	16 (73%)	10 (20%)	4 (8%)	1 (2%)	15 (31%)	31 (44%)

^1^Frequency (percentage of study participants for the category)

^2^Mean (standard error)

^3^Nutritional assessments for the participants’ intake of fruits and vegetables

**Table 2:** Study participants’ tumor characteristics.

	African American N = 22				Caucasian N = 49				
	TAC	TC	TCH	AA Total	TAC	TC	TCH	C. Total	Study Total
**Tumor Characteristic** ^**1**^	10 (46%)	6 (27%)	6 (27%)	22 (31%)	29 (59%)	15 (31%)	5 (10%)	49 (69%)	71 (100%)
**Luminal A** ^**1**^									
Yes	6 (27%)	2 (9%)	0	8 (36%)	21 (43%)	9 (18%)	0	30 (61%)	38 (54%)
No	4 (18%)	4 (18%)	6 (27%)	14 (64%)	8 (16%)	6 (12%)	5 (10%)	19 (39%)	33 (46%)
**Luminal B** ^**1**^									
Yes	0	0	2 (9%)	2 (9%)	2 (4%)	0	3 (6%)	5 (10%)	7 (10%)
No	10 (45%)	6 (27%)	4 (18%)	20 (91%)	27 (55%)	15 (31%)	2 (4%)	44 (90%)	64 (90%)
**Triple negative** ^**1**^									
Yes	3 (14%)	4 (18%)	1 (5)	8 (36%)	6 (1%2)	6 (12%)	0	12 (24%)	20 (28%)
No	7 (32%)	2 (9%)	5 (23%)	14 (64%)	23 (47%)	9 (18%)	5 (10%)	37 (76%)	51 (72%)
**HER2+, ER- & PR-** ^**1**^									
Yes	1 (5%)	0	3 (14%)	4 (18%)	0	0	2 (4)	2 (4%)	6 (8%)
No	9 (41%)	6 (27%)	3 (14%)	18 (82%)	29 (59%)	15 (31%)	3 (6%)	47 (96%)	65 (92%)
**Grade** ^**1**^									
1	1 (5%)	0	0	1 (5%)	2 (4%)	2 (4%)	0	4 (8%)	5 (7%)
2	5 (23%)	3 (14%)	4 (18%)	12 (55%)	12 (25%)	4 (8%)	0	16 (33%)	28 (39%)
3	4 (18%)	3 (14%)	2 (9%)	9 (41%)	15 (31%)	9 (18%)	5 (10%)	29 (59%)	38 (54%)
**Stage** ^**1**^									
I	1 (5%)	4 (18%)	0	5 (23%)	5 (10%)	8 (16%)	2 (4%)	15 (31%)	20 (28%)
IIA	6 (27%)	2 (9%)	3 (14%)	11 (50%)	11 (22%)	6 (12%)	1 (2%)	18 (37%)	29 (41%)
IIB	3 (14%)	0	3 (14%)	6 (27%)	5 (10%)	1 (2%)	2 (4%)	8 (16%)	14 (20%)
IIIA	0	0	0	0	8 (16%)	0	0	8 (16%)	8 (11%)

^1^Frequency (percentage of study participants for the category)

**Table 3:** Study participant treatments.

	African American N = 22				Caucasian N = 49				
	TAC	TC	TCH	AA Total	TAC	TC	TCH	C. Total	Study Total
**Treatment** ^**1**^	10 (45%)	6 (27%)	6 (27%)	22 (%)	29 (59%)	15 (31%)	5 (10%)	49 (%)	71 (100%)
**Surgery** ^**1**^									
Biopsy	3 (14%)	0	0	3 (14%)	2 (4%)	0	0	2 (4%)	5 (7%)
Lumpectomy	4 (18%)	0	1 (5%)	5 (23%)	8 (16%)	6 (12%)	1 (2%)	15 (31%)	20 (28%)
Segmental	1 (5%)	5 (23%)	3 (14%)	9 (41%)	1 (2%)	5 (10%)	0	6 (13%)	15 (23%)
Simple	2 (9%)	1 (5%)	2 (9%)	5 (23%)	18 (37%)	3 (6%)	4 (8%)	25 (52%)	30 (42%)
**Neoadjuvant** ^**1**^									
Yes	3 (14%)	0	1 (5%)	4 (18%)	2 (4%)	1 (2%)	0	3 (6%)	7 (10%)
No	7 (32%)	6 (27%)	5 (23%)	18 (82%)	27 (55%)	14 (29%)	5 (10%)	46 (94%)	64 (90%)
**Radiotherapy** ^**1**^									
Yes	9 (41%)	5 (23%)	5 (23%)	19 (86%)	23 (47%)	12 (24%)	1 (2%)	36 (73%)	55 (77%)
No	1 (5%)	1 (5%)	1 (5%)	3 (14%)	6 (12%)	3 (6%)	4 (8%)	13 (27%)	16 (23%)

^1^Frequency (percentage of study participants for the category)

Using the model building approach proposed by Hosmer and Lemeshow [44], a mixed effects linear model [45] was fit to determine the best subset of predictors of MN/cytome frequencies. In the first stage of the model building process, a base model was selected that represented the design of the data collection and of the timing of the treatments (chemotherapy and radiation). Fixed effects included visit (baseline, mid-chemo, six months and 1 year post-chemo), chemotherapy regimen, visit by chemotherapy interaction (chemotherapy was administered only during visit 2), radiation (Yes/No); visit by radiation interaction (radiation was only administered proximal to visit 3), and a random effect for study participant. In the second stage, each potential predictor was fit individually with the base model and, if the *p*-value was 0.25 or less, that predictor was used in the next stage. Potential predictors included demographic variables (age, race, BMI, income, menopausal status, alcohol consumption, and lifestyle nutrition), tumor characteristic variables (grade, stage. luminal A, luminal B, triple negative & HER 2 positive status), surgery, and PSS. In the third stage, all potential regressors (*p≤*0.25) were put into a multiple variable model. This initial model was further refined by sequentially removing variables from the model with the highest *p*-values (backward stepwise) until all remaining factors had a *p*-value of 0.05 or less. At this stage, all pairwise interactions were added. Again using a backward stepwise approach, all interaction terms with *p*-values greater than 0.05 were removed. This model was considered the final prediction model. The SAS v9.4 and JMP v11.1 statistical packages were used for these analyses (SAS 9.3 and JMP 11.1: SAS Institute Inc., SAS Campus Drive, Cary, North Carolina 27513).

## Results

A total of 77 women were recruited for this study, with only 3 women (all Caucasians; ages 48, 62, and 66) failing to complete the multiple visits required for this longitudinal investigation, which resulted in a 96% retention rate. Two of the 3 women who did not complete the study elected to withdraw due to feeling “overwhelmed”. The third woman, who developed osteomyelitis after visit 2, no longer met the eligibility criteria and thus was excluded from the study. Data and specimen collections were completed for visits 1 (baseline) and 2 (mid-chemo) for all 74 retained study participants. However, MN/cytome abnormality frequencies were not successful at baseline or visit 2 for one woman, reducing the sample size to 73 participants. At the time of data analysis in this ongoing study, 64 of the 73 participants had completed visits 1, 2, and 3 (visit 3 occurs 6 months post the initiation of chemo), and 50 women had completed visits 1, 2, 3 and 4 (visit 4 occurs 1 year after chemo initiation). MN/cytome abnormality frequencies were successfully obtained for all visit 3 and 4 specimens.

### Chemotherapy and Radiation Treatments

Three primary types of chemotherapy regimens were administered to the study participants. These were categorized as: (1) TAC, which included women who received sequential administration of doxorubicin (Adriamycin), cyclophosphamide (Cytoxan), and docetaxel (Taxotere); (2) TC, which included women who received docetaxel (Taxotere) and cyclophsophamide (Cytoxan); or (3) TCH, which included women who received docetaxel (Taxotere), carboplatin (Paraplatin), and trastuzumab (Herceptin). Of the 73 women fully participating in this study, the majority (n = 39) of participants received a TAC chemotherapy regimen. A total of 21 participants received TC treatment, and 11 women received TCH treatment. Two study participants received a cyclophosphamide, methotrexate and 5-fluorouracil treatment. Given the small number of women receiving this latter treatment, these two participants were excluded from the statistical analysis. Thus, comparisons of exposure factors influencing peripheral blood cell chromosomal instability frequencies were completed for a total of 71 women.

Of these 71 women, a total of 55 participants received radiotherapy (77%), including 32 of the 39 women assigned to the TAC regimen, 17 of the 21 assigned to the TC regimen, and 6 of the 11 women assigned to the TCH regimen.

### Demographic and health information

Demographic data for the 71 participants receiving the most common types of treatment regimens is shown in Table 1. These participants included 22 African American and 49 Caucasian women. Two women (1 in the African American cohort and 1 in the Caucasian cohort) self-reported that they were of Hispanic ethnicity. However, given the small number of women having a Hispanic heritage, this sub-group was not analyzed separately.

The average age of the study participants was 51.3 years, with a significant difference in age being observed between the African American (mean = 46.6 years, s.e. = 1.9 years) and Caucasian (mean = 53.5 years, s.e. = 1.5 years) sub-groups (*p* = 0.0079) (Table 1). Annualized income levels also varied between the racial groups, with the Caucasian patients having significantly higher incomes than the African American women (*p*<0.0001). As expected, the majority of women (56%) were post-menopausal with 44% being either pre- or peri-menopausal (Table 1). No significant difference in menopausal status was detected between the African American and Caucasian study participants, but (as expected due to the younger age of the African American women) a trend was observed toward more pre- or peri-menopausal women in the African American cohort (*p* = 0.2163). Comparisons of lifestyle/exposure histories showed that significantly more Caucasian women reported consuming alcohol [69% (34/49)] when compared to African American women [27% (6/22)](*p* = 0.0016)(Table 1). None of the 14 women who reported smoking met the criteria of a heavy smoker (≥ 30 cigarettes per day), as suggested by Fenech et al. [46]. Thus, smoking was not included in the stepwise prediction model.

### Tumor Characteristics

Due to the inclusion criteria established for this study, all of the study participants had early stage breast cancer (stages I to IIIA). The participants’ tumor characteristics and their pre-chemotherapy treatments are summarized in Tables 2 and 3, respectively. While the proportion of African American women having grade 3 tumors (41%) tended to be lower than the proportion for Caucasian women (59%), the grade distributions of the tumors were not significantly different between races (*p* = 0.153). Similarly, while no significant difference in the overall proportion of tumors subtypes was observed between the racial sub-groups (*p* = 0.5698), there was a trend toward a higher proportion of triple negative or HER2+ tumors and a lower proportion of luminal A tumors in the African American women (36% triple negative tumors; 18% HER2+; 36% luminal A tumors) compared to the Caucasian women (24% triple negative tumors; 4% HER2+; 61% luminal A tumors; (Table 2). A significant difference in the surgical approaches used for African American compared to Caucasian women was observed (*p* = 0.0135), but no differences in adjuvant/neoadjuvant status (*p* = 0.1305), chemotherapy (chemotherapy *p* = 0.2011) or radiotherapy (*p* = 0.213) regimens were observed between the racial groups (Table 3).

### Perceived Stress Scores (PSS)

An assessment of total PSS showed a higher value at baseline for the total group of study participants (pooled across all women) (Table 4) (*p* = 0.0024). The proportion of women reporting “high/severe” total PSS values (as defined in the DASS21-stress subscale; [47] ranged from 20% at baseline to 8% 1 year following the initiation of chemotherapy (Table 4). A total of 6 women (2 African American women and 4 Caucasian women) reported high perceived stress levels across more than one visit (range of 2 to 4 visits), with the remaining high PSS values being associated with a single time point (either visit 1, 2, 3, or 4).

**Table 4:** Perceived Stress Levels Reported by Women Treated for Breast Cancer.

	Total Perceived Stress (PSS-10)^1^								
	Normal (0–15)			Mild to Moderate (16–22)			High/Severe (23–40)		
Visit	N^2^	Mean	Std Err	N^2^	Mean	Std Err	N^2^	Mean	Std Err
1	32	10.06	0.74	25	19.00	0.44	14	28.43	1.46
2	38	8.84	0.65	21	18.56	0.43	11	26.55	0.72
3	38	8.82	0.72	18	19.22	0.50	8	28.00	1.51
4	30	8.23	0.94	15	18.20	0.45	4	26.25	1.80

^1^The DASS21 descriptors/PSS-10 correlate values are based on Andreou, et al., 2011.

^2^N = Number of women providing values for this PSS-10 score category. PSS-10 values were not available for one women at visit 2 (case 2076) and one woman at visit 4 (case 2005).

### NDCI values

To determine if there might be differential levels of cellular proliferation/viability following exposure to the diagnosis and treatment of breast cancer, NDCI values were evaluated (Table 5). No significant differences in NDCI values were observed with visits, chemotherapy or radiotherapy.

**Table 5:** Nuclear division cytotoxicity index (NDCI) values in lymphocytes.

Visit	Chemotherapy Regimen					
	TAC		TC		TCH	
	Mean	Std Err	Mean	Std Err	Mean	Std Err
1	1.89	0.05	1.88	0.09	1.88	0.08
2	1.93	0.05	1.94	0.07	1.83	0.07
3	2.03	0.05	2.01	0.12	1.84	0.17
4	1.86	0.06	1.87	0.03	1.87	0.03

### Model Fitting to Identify Factors Predictive of MN/Cytome frequencies

To determine if any of the treatment, health, stress, or demographic variables were associated with MN/cytome abnormality frequencies, the data were fit to a mixed effects linear model. In the second stage, each potential predictor was fit individually with the base model and, if the *p*-value was 0.25 or less, that predictor was used in the next stage. Potential predictors fit included: age (*p* = 0.9599), race (*p* = 0.0652), BMI (*p* = 0.2719), income (*p* = 0.3728), menopausal status (*p* = 0.4964), alcohol consumption (*p* = 0.0816), lifestyle nutrition (*p* = 0.3062), grade (*p* = 0.7563), stage (*p* = 0.2981), luminal A (*p* = 0.9807), luminal B (*p* = 0.0594), triple negative (*p* = 0.1792), HER 2 positive status (*p* = 0.6460), surgery (*p* = 0.3042), and Perceived Stress Scale (PSS)(*p* = 0.1088).

**Fig 2:**
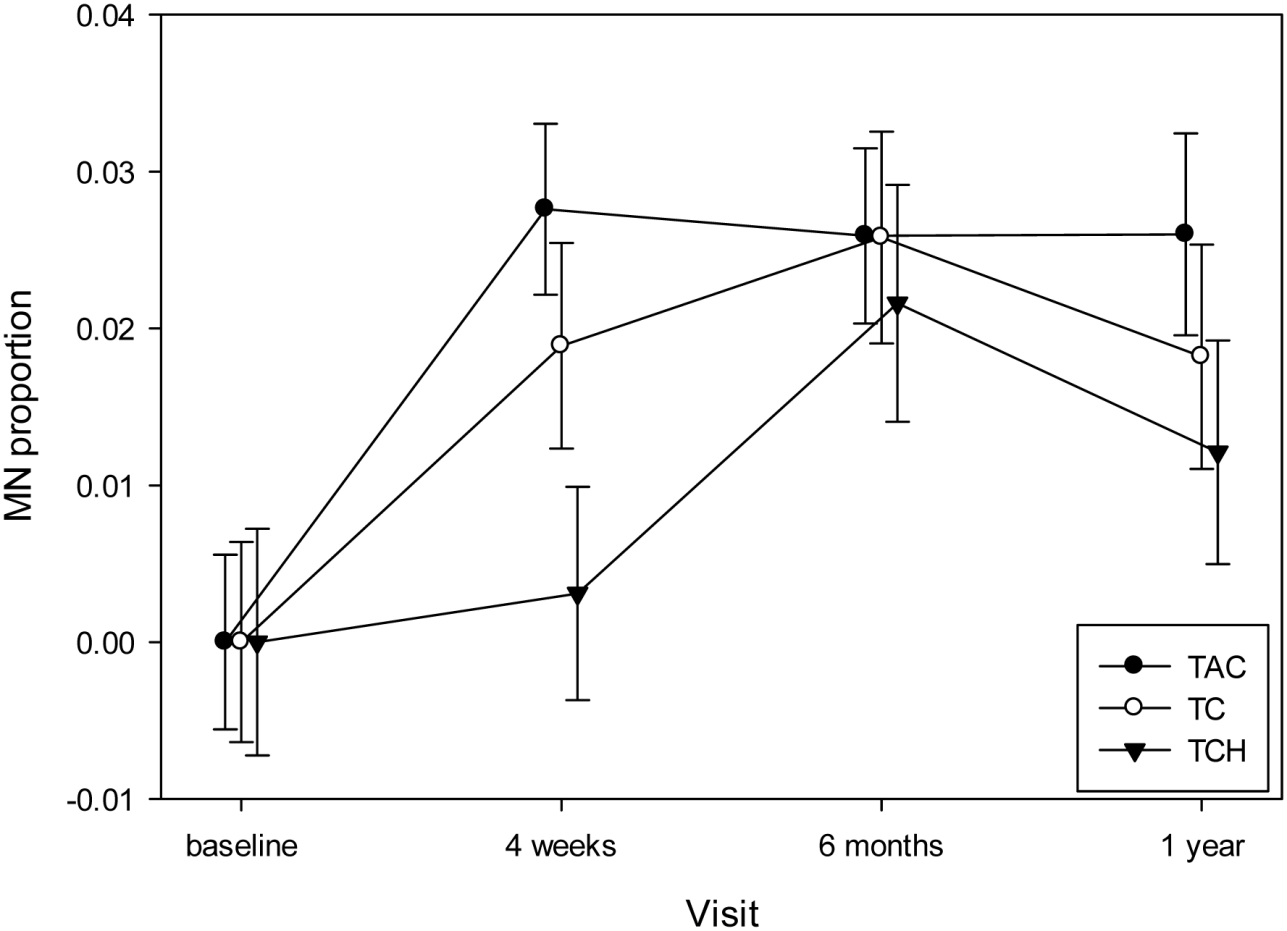
Changes in MN/cytome abnormality frequencies over the 1 year follow-up period based on the women’s chemotherapy regimen. When compared to baseline values, the frequencies of acquired chromosomal instability values were significantly higher for at least 1-year following the initiation of treatment (visit 4) for the women who received the TAC (*p*<0.0001) or TC (*p* = 0.014) regimens, but not the TCH treatment (*p* = 0.0884). TAC = black circles; TC = white circles; TCH = black triangles.

**Fig 3:**
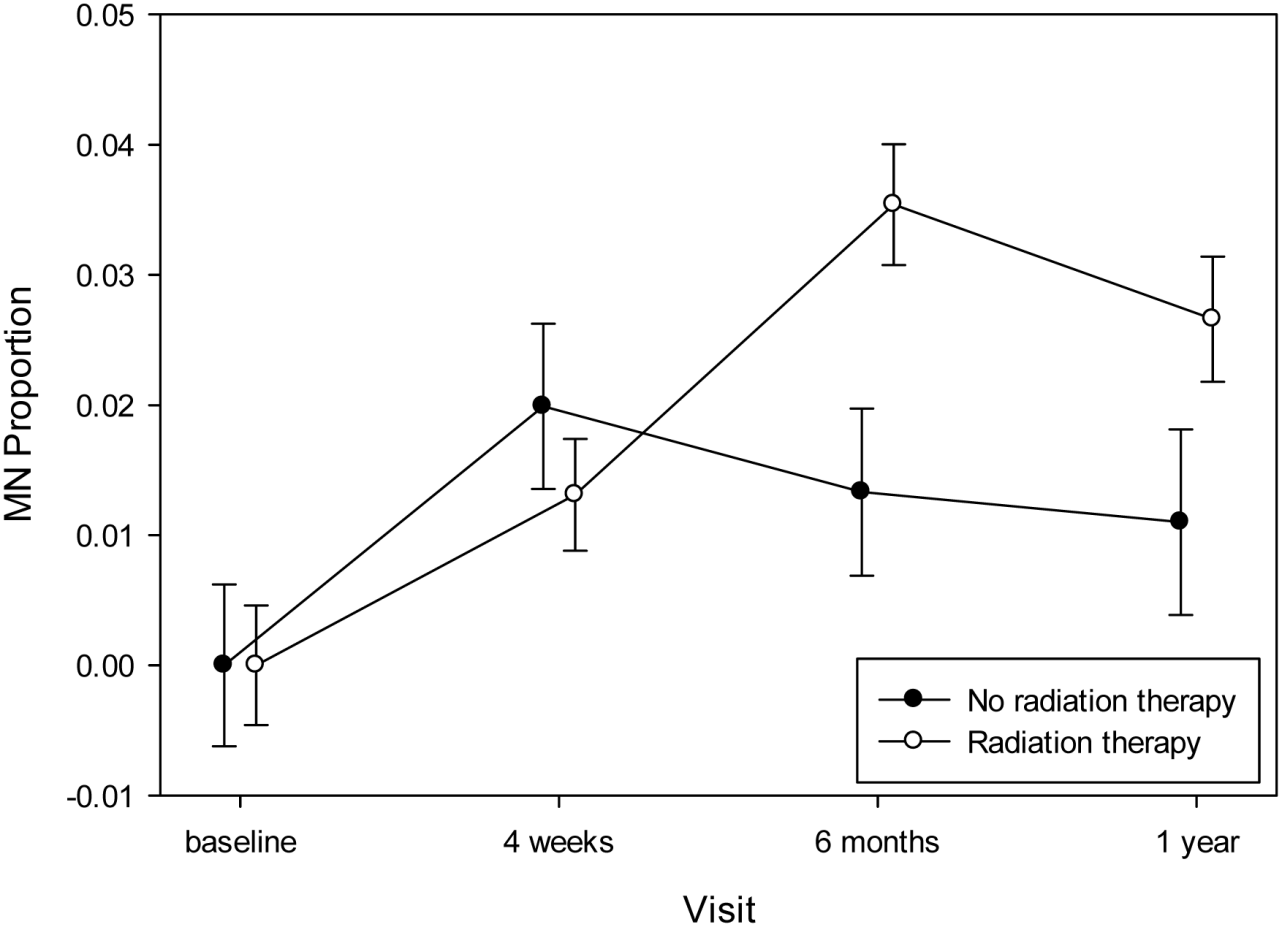
Changes in MN/cytome abnormality frequencies associated with radiotherapy. At the baseline and 4 weeks (mid-chemo) time points, no significant differences in the chromosomal instability frequencies were observed between the women who did (white circles) or did not (black circles) receive radiation therapy. However, a statistically significantly increase in MN/cytome abnormality frequencies was observed at six months (compared to baseline) for the group of women receiving radiotherapy versus the group not receiving radiation (see Table 6). Also, for the subset of women who received radiotherapy, this increase in chromosomal instability values persisted at the 1 year time point (p<0.0001).

The final model is reported in Table 6 and showed significant effects attributable to a subset of items in the base model, as well as effects for race (*p* = 0.0061), luminal B tumors (*p* = 0.0053) and PSS (*p* = 0.0129). When assessing the effect of chemotherapies on somatic chromosomal instability levels, at the mid-chemo time point (scheduled prior to the fourth treatment [visit 2]) the women who received TAC chemotherapy showed the greatest increase in MN/cytome abnormality frequencies when compared to baseline values (*p* = 0.0329) (Fig 2). Also, the mean MN/cytome abnormality frequency was noted to be significantly lower at baseline than at visit 4 for the women who received TAC (*p*<0.001) or TC (*p* = 0.014) regimens, but not the TCH treatment (*p* = 0.0884). An impact of radiotherapy on MN/cytome abnormality frequencies was demonstrated by a significant increase in chromosomal instability levels one year post the initiation of chemo [visit 4] compared to baseline [visit 1] in the women receiving radiotherapy (*p*<0.0001)(Fig 3). Overall, a significant change in chromosomal instability frequencies was observed over time, with this change differing based on whether the women received radiotherapy (Radiation by Visit; p = 0.0052)(Table 6).

**Table 6:** Mixed effects linear model fitting assessment of predictive associations of variables with peripheral blood micronuclei/cytome abnormality frequencies^1^ ^,^ ^2^.

Variable	NDF	DFD	F Ratio	p-value
Visit	3	175.1	12.7033	<.0001
Chemo	2	63.58	2.1494	0.1249
Radiation	1	65.05	7.3550	0.0085
Chemo by Visit	6	174.1	0.9863	0.4361
Radiation by Visit	3	175.7	4.3921	0.0052
Race	1	61.48	8.0619	0.0061
Luminal B	1	67.08	8.3236	0.0053
PSS	1	145.6	6.3448	0.0129

^1^The base model, which was determined by the study design, was: MN frequency = Visit + Chemotherapy (3 types) + Radiation therapy + Visit by Chemotherapy* + Visit by Radiation therapy* with the study subject being a random effect

^2^Variables having predictive *p* values greater than 0.25 in the initial stepwise model included: Age (p = 0.9599); BMI (*p* = 0.2719); Income (*p* = 0.3728); Menopausal status (*p* = 0.4964); Alcohol consumption (*p* = 0.0816); Tumor grade (*p* = 0.7563); Tumor stage (*p* = 0.2981); Luminal A (*p* = 0.9807); HER2 positive (*p* = 0.6360); and Surgery (*p* = 0.3092)

*The visit by chemo and visit by radiation interaction variables allowed these values to differ across time points.

**Fig 4:**
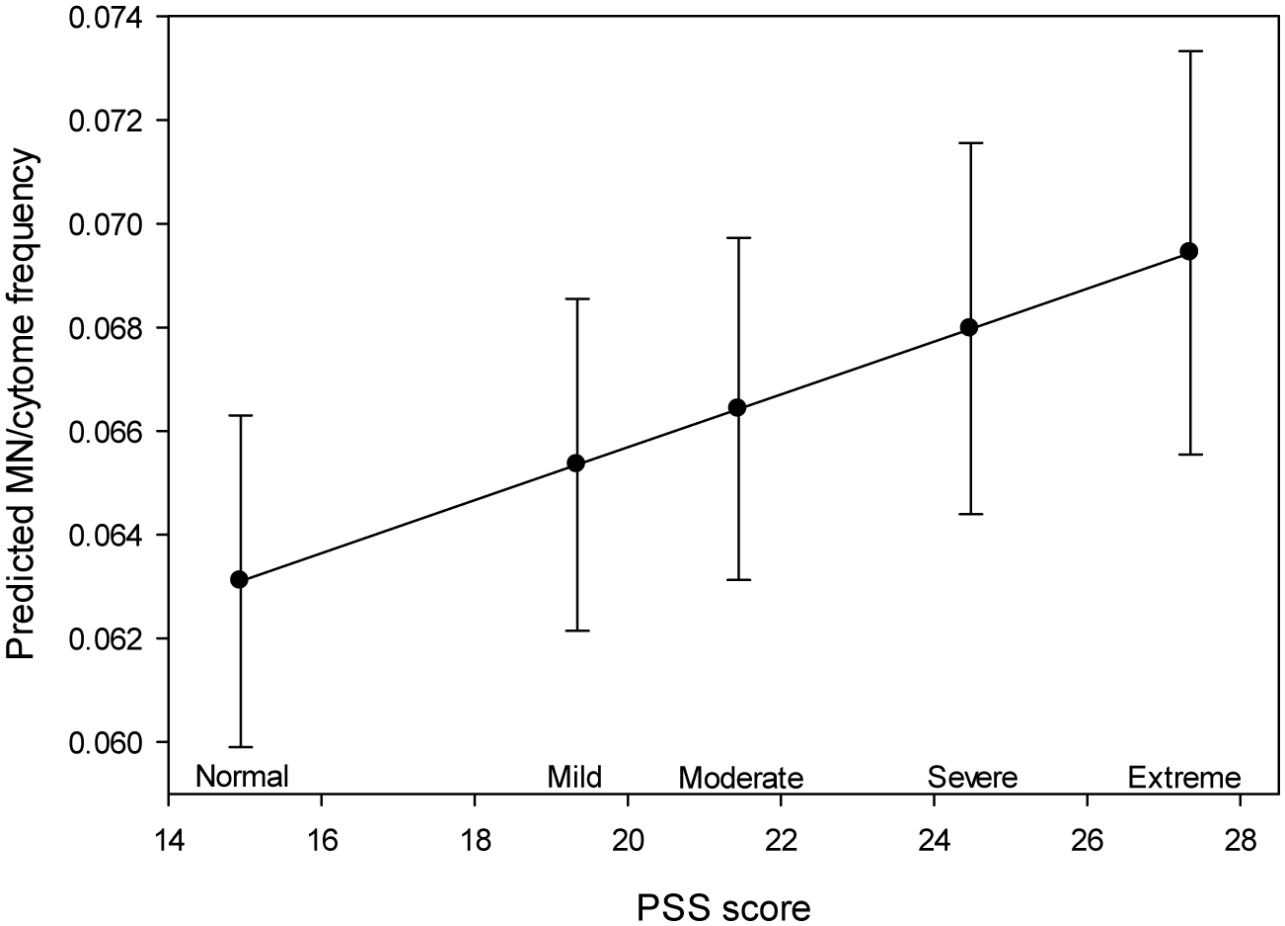
Increased MN/cytome abnormality frequencies were significantly associated with higher levels of perceived stress. The results of the linear mixed effects model are illustrated in this figure, which shows the increasing trend line of the MN/cytome frequency with increasing PSS (p = 0.0129). The PSS categories proposed by Andreou, et al., 2011 are shown on the X axis (a subset of PSS range); the MN/cytome frequencies (predicted values from linear mixed effects model) are shown on the Y axis.

Acquired somatic cell chromosomal instability was also observed to be significantly impacted by factors other than those directly attributable to therapy. The influence of perceived stress on MN/cytome frequencies was detected for the total data set (across all visits), and was not differentially associated with a specific diagnostic, therapeutic or recovery time point in this longitudinal study (Fig 4). Tumor characteristics (specifically luminal B tumors) were also shown to be predictive of MN/cytome abnormality frequencies. Given that the tumor characteristics determine the type of therapy regimen received by the study subjects, it is feasible that this predictive relationship is confounded by the impact of chemotherapy and/or radiation. However, the retention of a consistent significant association for tumor characteristic variables and MN/cytome abnormality frequencies, following the stepwise removal of the potentially related therapy variables, suggest the presence of a separate effect [distinct from the effect(s) related to therapies] for the tumor sub-types on peripheral blood MN/cytome abnormality frequencies. Lastly, race was also observed to be predictive of chromosomal instability frequencies (*p* = 0.0061). Specifically, MN/cytome values were significantly lower in the African American participants (mean = 0.059; SE = 0.0043) compared to the Caucasian participants (mean = 0.071; SE = 0.0031). One might conjecture that this association is confounded by other influences (e. g. age differences between the racial sub-groups; tumor attribute variation between races). However, as noted above, the consistent recognition of an association between MN/cytome abnormality frequencies and race following the stepwise removal of other potential confounding variables (including age and tumor characteristics) supports the presence of a separate, differential impact of race on chromosomal instability frequencies following treatment for breast cancer.

## Discussion and Conclusions

By evaluating MN/cytome frequencies using a longitudinal study design we identified exposure-related, tumor-related, and intrinsic factors that were predictive of acquired chromosomal instability levels in lymphocytes. These predictive factors included: (1) the time interval during the course of diagnosis, treatment, and recovery (e.g. visit); (2) the type of chemotherapy received; (3) whether radiotherapy was received; (4) the woman’s perceived stress level; (5) tumor attributes; and (6) race.

### Visit

The MN/cytome frequencies were lowest at baseline values, with the values observed 1 year after the initiation of therapy being higher than baseline values. The highest frequencies of chromosomal instability were observed for visits 2 and 3, which coincide with the mid-cycle of chemotherapy administration (100% of women) and radiotherapy administration (77.5% of women), respectively. This visit-related observation is not surprising given that both of these types of treatment have been shown to be genotoxic and/or have the potential to induce DNA/chromosomal damage [48].

### Chemotherapy

The lymphocytes from most women showed increases in chromosomal instability during the administration of chemotherapy. Each of the three chemotherapeutic regimens administered to the study participants included the drug docetaxel (Taxotere). Given that this drug causes microtubule depolymerization, one could anticipate that it might contribute to the acquisition of chromosomal abnormalities, especially aneuploidy [49]. Of the three chemotherapeutic regimens administered to women in this study, the frequency of MN/cytome abnormalities was highest for the TAC regimens at the mid-cycle time (visit 2). Doxorubicin is the likely contributory factor to this observed effect since this is the only drug that differs between the TC and TAC regimens. Doxorubicin is a member of the anthracyclin class of compounds. Although this drug is widely used, the mechanisms for its effectiveness in preventing/reducing cancer cell proliferation are not fully known, with some investigators proposing that it induces cell apoptosis or senescence by inhibiting topoisomerase II, while others cite roles for DNA adduct formation, oxidative stress, ceramide overproduction and/or torsion-induced nucleosome destabilization as the causal means for this drug’s efficacy [50–53]. Although the mechanisms of action for doxyrubicin remain controversial, this drug has consistently been shown to induce chromosomal instability (Table 7), with telomere dysfunction also being associated with this increase in acquired chromosomal abnormality frequencies [54–56]. Thus, the results of our longitudinal *in vivo* study are in agreement with the *in vitro* findings of several other investigators and the *in vivo* results of the few other groups reporting frequencies following treatment in humans (Table 7) [57–67].

**Table 7:** Summary of previous findings related to cancer/cancer treatment in which MN frequencies were evaluated in peripheral blood.

Topic	References
**Breast Cancer Effects**	
Higher MN frequencies at baseline for women with breast cancer prior to treatment irrespective of smoking and aging	Santos et al., 2010 [57]
Significant differences in MN frequencies at baseline between patients with breast cancer and controls	Cardinale et al. 2012 [58]
**Chemotherapy Effects**:	
*In vitro* study showing variation in response to doxyrubicin-induced MN between lymphocyte cell lines; Polymorphism in the GSTP1 gene was associated with an increased MN frequency	Ramos et al., 2011 [59]
*In vitro* study showing a significant increase in MN frequencies in breast cancer cell lines following exposure to doxyrubicin; use of both methoxyamine and doxorubicin significantly increased levels of MN frequencies (combination higher than single exposure values)	Guerreiro et al., 2013 [60]
*In vivo* study showing MN frequencies in lymphocytes and buccal mucosa cells from children with malignant tumors (various types) increased significantly following chemotherapy	Minicucci et al., 2008 [61]
*In vivo* longitudinal study (1 year) showing MN frequencies increased significantly after one cycle of chemotherapy and remained increased for 2 months after the completion of chemotherapy (Cisplatin); MN frequencies also correlated with the cumulative dose of Cisplatin and with Cisplatin-induced nephrotoxicity	Elsendoorn et al., 2001 [62]
**Radiation Effects**:	
*In vivo* study showing MN frequencies increased significantly during radiation and further increased immediately following the completion of radiation; MN frequencies not influenced by age.	Jagetia et al., 2001 [63]
Higher MN frequencies during radiotherapy; while decreases in frequency were observed 6 months and one year after radiotherapy, MN frequencies did not return to pre-therapy levels for the majority of patients; older subjects (ages 75–80; also had more advanced tumors) showed higher MN frequencies than younger subjects (ages 57–70).	Gamulin et al., 2010 [64]
*In vivo* studies showing radiation-induced MN frequencies were correlated with dose and quality of occupational, medical, accidental, or therapy-related radiation.	Vral et al., 2011 [65]
*In vitro* study showing significant increase in MN frequencies in cells exposed to ionizing radiation, with the difference in frequency responses of patients with breast cancer compared to controls exceeding the differences observed at baseline.	Cardinale et al., 2012 [58]
**Chemotherapy and Radiation Effects**:	
*In vivo* study showing an increased frequency following radiation alone and following radiation in combination with chemotherapy regardless of age or smoking.	Milosevic-Djordjevic et al., 2011 [66]
*In vivo* longitudinal study (18 months) showing MN frequencies increased after radiation (20 patients received radiation) but not after chemotherapy (only 9 out of 20 patients received and were assessed after chemotherapy); MN frequencies were not significantly correlated with tumor size, receptor status, nodal status, family history of cancer, age, or smoking habits.	Aristei et al., 2009 [67]

The women receiving the TAC regimen also had higher chromosomal instability values than the women receiving the TCH regimen, the latter of which includes carboplatin and trastuzumab. Carboplatin is an alkylating agent that causes cross-linking of guanine bases in DNA, which in turn, leads to supercoiling of DNA, thereby compromising DNA separation, replication and division [68]. Trastuzumab, which is a targeted monoclonal antibody that binds to the HER2 receptor, has been associated with multiple influences on cells that prohibit their proliferation, including (but not limited to), the arrest of cells in the G1 phase of the cell cycle, disruption of signaling cascades (PI3K), suppression of angiogenesis, and the facilitation of immune cell-mediated cytotoxicity [69–71].

The total body clearance for these chemotherapeutic drugs (TC, TAC, and TCH regimens) has been shown to be influenced by BMI and age [72–75]. However, our study results showed no impact of BMI or age on MN/cytome frequencies. Thus, one can infer that the persistence of somatic chromosomal instability (at least 1 year following chemotherapy treatment) reflects the presence of cells/clonal cell lines that originated at the time of exposure to these drugs. Alternatively, the long-term increased MN/cytome frequencies may reflect additional influences beyond direct temporal exposure to the chemotherapeutic regimens since the drug exposure for the TC and TAC regimens was completed by visit 3 (women receiving the TCH regimen continue to receive trastuzumab for up to one year). Also, our observation of persistence of acquired chromosomal instability frequencies in lymphocytes for at least one year following cancer treatment underscores the potential impact that these therapeutic regimens have on all types of somatic cells (healthy and cancerous).

### Radiotherapy

Akin to the impact of chemotherapy, radiotherapy was also identified to have a significant predictive influence on the women’s acquired peripheral blood chromosomal instability frequencies. As with Doxorubicin, radiation exposure has been consistently associated with increased levels of chromosomal instability in human cells, primarily through *in vitro* study designs (Table 7).

### Perceived Stress

In addition to the treatment-related exposures, women diagnosed with breast cancer experience considerable stress. While no significant differences in stress levels were reported across the 4 visits assessed over the one year interval evaluated in this study, levels at baseline (visit 1) tended to be highest and those at visit 4 tended to be lower than visits 2 and 3. This same pattern in perceived stress levels was also observed in 227 women with breast cancer who were evaluated by Wu, et al. [76] as part of a 1 year longitudinal study to assess physiological stress responses.

To our knowledge, this is the first report of a significant predictive positive association between perceived stress levels and acquired chromosomal instability in lymphocytes from women longitudinally studied following their treatment for breast cancer. York et al. [26] were the first to show a significant association between stress and an increased rate of peripheral blood MN/cytome abnormality frequencies through their studies of identical twins who were discordant for exposure to childhood sexual abuse. In agreement with these observations, Morath, et al. [77] recently showed that DNA breakage occurred at an increased frequency in people with post-traumatic stress disorder. Analogous to these human studies, research using a mouse model has shown high stress to be associated with an increased susceptibility to the mutagenic effects of radiation and drug exposure, leading to increased levels of chromosomal aberrations and MN in mice with higher stress [78]. Therefore, our observation of a predictive relationship between perceived stress related to breast cancer and its treatment is in agreement with the few previous reports assessing relationships between stress and chromosomal/genome instability.

While there is a paucity of studies investigating associations between stress and acquired chromosomal instability frequencies in women with breast cancer (or any type of cancer), several investigators have reported an association between stress and telomere shortening [27, 79]; especially chronic stress [80]. Given that telomere shortening has also consistently been observed to result in increased frequencies of acquired chromosomal abnormalities, it follows that stress may also be associated with chromosomal instability.

### Race

An unexpected finding in this study was the significant predictive association of race on MN/cytome abnormality frequencies, with the Caucasian study participants having higher overall instability frequencies than the African American participants. While our step-wise statistical approach suggests this observation is an independent association, given the inter-relatedness of human epidemiology variables, one cannot fully rule out the possibility that it may be confounded by other factors, such as age or tumor characteristics. For example, the Caucasian women participating in this study were significantly older than the African American participants. This difference is consistent with data from the American Cancer Society, which shows that African American women develop more aggressive forms of breast cancer at younger ages and show a poorer prognosis than Caucasians. Given that increasing age has consistently been shown to be positively correlated with MN frequencies in healthy women [30, 81], it is possible that the age of the Caucasian participants contributed to their observed increased rate of MN/cytome abnormalities. We also observed differences in annualized income levels and alcohol consumption rates between the racial groups in our study, with the Caucasian women having significantly higher incomes and alcohol consumption practices than the African American women. Therefore, given the inter-related nature of the data, the basis for the association between race and MN/cytome abnormality frequencies in this study is not clear, but warrants further study.

There is a paucity of information available regarding MN frequencies in healthy subjects from different racial groups [82] and to our knowledge, there are no previous reports of lymphocyte MN/cytome frequency differences of racially diverse women treated for breast cancer. However, the observation that spontaneous somatic mosaicism involving large (greater than 2 megabases) autosomal alterations occurs more frequently with age (consistent with observations of spontaneously occurring MN in healthy people), but less frequently in people of African ancestry compared to European ancestry, suggests that there may be differences in acquired somatic cell chromosomal instability levels that are related to race [83]. It is interesting to note that African American women have been described to have significantly lower levels of dietary-related oxidative DNA damage than white women, suggesting that there may also be race-related differences in the cellular processes involved in oxidative DNA damage recognition/repair [84]. Moreover, ethnic differences have been detected for chemotherapy drug metabolism and inflammatory responses to cancer treatment [85], both of which might contribute to the acquisition of somatic cell chromosomal instability.

### Tumor characteristics

Another aspect of breast cancer that has been associated with racial differences is the proportion of breast cancer tumor sub-groups in African American compared to Caucasian populations [86]. We observed a trend toward a higher proportion of HER2 positive (18%) tumors in the African American participants in our study compared to those noted in the Caucasian participants (4%). This trend is consistent with the pattern reported by Carey, et al [86] who studied 496 women from North Carolina. Thus, the breast tumors in our participants appear to be representative of those seen in larger populations. The treatment regimen for each study participant was determined, in large part, by her breast tumor characteristics. Therefore, if treatments are related to chromosomal instability levels, one might also anticipate detecting the presence of a relationship between tumor characteristics and acquired chromosomal changes. However, our stepwise reduction model showed a predictive influence of tumor characteristics on MN/cytome abnormality frequencies for the subset of women having luminal B tumors beyond that of treatment. Differential response in the gene expression patterns of luminal compared to basal epithelial cells following exposure to chemotherapeutic agents has been reported [87] and it is feasible that the gene expression differences could impact acquired chromosomal instability levels in healthy normal tissues. Another observation that supports that conjecture that the actual tumors (not just treatments) can influence acquired chromosomal instability levels in peripheral blood comes from reports of consistently higher pre-treatment MN frequencies in women and men with a variety of cancers, especially when compared to age-matched controls without cancer [30, 81].

### Age Effect

We did not detect a predictive association between age and MN/cytome abnormality frequencies in our women receiving treatment for breast cancer. Other investigators have also failed to detect an age effect for MN frequencies in patients with cancer, or specifically with breast cancer, despite the clear association between age and MN frequencies that has been observed in healthy adults [30, 81]. This lack of a clear, independent influence of chronological age on peripheral blood cell MN frequencies in women with breast cancer could reflect the narrow age range of the participants sampled in this study (and other studies of women with breast cancer). Alternatively, the influence(s) of factors related to breast cancer development may override or “mask” the effects of age.

### Other factors

In addition to the exposure-related factors evaluated in this study, the frequency of acquired chromosomal instability in lymphocytes following treatment for cancer is likely to reflect germ-line genetic make-up differences between people, which are factors that were not evaluated in this study. For example, pharmocogeneticists have shown differential metabolism of chemotherapy drugs based on the variant form(s) of alleles that an individual carries for several genes (e. g. CYP2D6, ESR1, ESR2, etc), including genes involved in DNA repair (FANCD2; MTHFR, RB1) [88–91]. Furthermore, in healthy people, baseline levels of micronuclei have been shown to be influenced by heritable genetic (as well as environmental) factors, including polymorhisms of genes involved in DNA repair [92, 93].

### Conclusions

In summary, we determined that women treated for breast cancer showed higher lymphocyte chromosomal instability frequencies during and after treatment than at baseline, with these increased frequencies persisting for at least one year following treatment. Of particular interest was the recognition of stress as a contributor to increased levels of chromosomal instability, since this factor has the potential to be modulated (reduced) through intervention. Given that stress-related chromosomal instability has been shown to persist (and even accumulate) for years [26], one could speculate that these somatic chromosomal changes could lead to gene expression imbalances that could contribute to the development/persistence of long-term side effects associated with treatment for cancer. Alternatively, the increases in peripheral blood MN/cytome abnormality frequencies may be simply correlative in nature rather than mechanistically related, with the frequency of chromosomal instability in healthy cells serving as a “chronicle” or biomarker to document that biologically relevant soma-wide changes have occurred that could lead to PNS. Regardless of the direct or indirect role played by acquired chromosomal instability, this MN/cytome test warrants further study to determine if it can be used as a potential biomarker for the future development of an algorithm to identify individuals most “at risk” for acquiring persistent adverse side effects associated with cancer and to determine if interventions to reduce stress could have a positive health outcome on women with increased levels of chromosomal instability following their treatment for breast cancer.

## Supporting Information

S1 Table Summary data for measures evaluated in this manuscript. (XLSX)
